# Adapting domestic abuse training to remote delivery during the COVID-19 pandemic: a qualitative study of views from general practice and support services

**DOI:** 10.3399/BJGP.2022.0570

**Published:** 2023-06-13

**Authors:** Elizabeth Emsley, Eszter Szilassy, Anna Dowrick, Sharon Dixon, Anna De Simoni, Lucy Downes, Medina Johnson, Gene Feder, Chris Griffiths, Jasmina Panovska-Griffiths, Estela Capelas Barbosa, Vari Wileman

**Affiliations:** Bristol Medical School, University of Bristol, Bristol.; Bristol Medical School, University of Bristol, Bristol.; Nuffield Department of Primary Care Health Sciences, University of Oxford, Oxford.; Nuffield Department of Primary Care Health Sciences, University of Oxford, Oxford.; Wolfson Institute of Population Health, Queen Mary University of London, London.; Identification and Referral to Improve Safety network director;; Identification and Referral to Improve Safety interventions, Bristol.; Bristol Medical School, University of Bristol, Bristol.; Wolfson Institute of Population Health, Queen Mary University of London, London.; Pandemic Sciences Institute and Big Data Institute, University of Oxford, Oxford.; School of Policy and Global Affairs, City, University of London, London.; School of Mental Health and Psychological Sciences, Institute of Psychiatry and Neuroscience, King’s College London, London.

**Keywords:** COVID-19, domestic violence, general practice, qualitative research, SARS-CoV-2, training activities

## Abstract

**Background:**

Identifying and responding to patients affected by domestic violence and abuse (DVA) is vital in primary care. There may have been a rise in the reporting of DVA cases during the COVID-19 pandemic and associated lockdown measures. Concurrently general practice adopted remote working that extended to training and education. IRIS (Identification and Referral to Improve Safety) is an example of an evidence-based UK healthcare training support and referral programme, focusing on DVA. IRIS transitioned to remote delivery during the pandemic.

**Aim:**

To understand the adaptations and impact of remote DVA training in IRIS-trained general practices by exploring perspectives of those delivering and receiving training.

**Design and setting:**

Qualitative interviews and observation of remote training of general practice teams in England were undertaken.

**Method:**

Semi-structured interviews were conducted with 21 participants (three practice managers, three reception and administrative staff, eight general practice clinicians, and seven specialist DVA staff), alongside observation of eight remote training sessions. Analysis was conducted using a framework approach.

**Results:**

Remote DVA training in UK general practice widened access to learners. However, it may have reduced learner engagement compared with face-to-face training and may challenge safeguarding of remote learners who are domestic abuse survivors. DVA training is integral to the partnership between general practice and specialist DVA services, and reduced engagement risks weakening this partnership.

**Conclusion:**

The authors recommend a hybrid DVA training model for general practice, including remote information delivery alongside a structured face-to-face element. This has broader relevance for other specialist services providing training and education in primary care.

## INTRODUCTION

Domestic violence and abuse (DVA) is a violation of human rights challenging global public health policy and clinical practice. DVA damages mental and physical health for victims and their children.^1^ Although DVA can affect everyone, the experience of DVA is gendered, with research often focusing on women.^2^ Women experiencing DVA have increased health service use and are more likely to contact health services than those unaffected by abuse.^3^ In general, patients attending general practice also have a higher lifetime prevalence of DVA compared with the wider population.^4^^–^^6^ Therefore, the general practice response to DVA is crucial in supporting patients and provides a vital link to specialist services. During the COVID-19 pandemic, there was a rise in reporting of DVA cases occurring alongside a transition to remote general practice consulting.^7^^,^^8^ Remote working also extended to training and education, including DVA training.^9^

IRIS (Identification and Referral to Improve Safety) is a DVA training, support, and referral programme developed by IRIS interventions (IRISi)^10^ and delivered by local DVA services. IRIS transitioned to remote delivery during the pandemic. DVA training for the whole general practice team is part of a broader intervention aiming to establish a referral pathway between general practice and DVA support services. Advocate educators are specialist DVA workers in the IRIS programme.^11^ The advocate educator role includes training general practices and receiving patient referrals for expert advocacy.^11^ The IRIS programme was trialled in 2007–2008,^12^ and was recommended in high-level UK government documents, with over 1275 general practices having received training.^13^^–^^16^ However, challenges have included difficulties in scheduling training and maintaining general practice engagement.^17^ General practice engagement is essential when building a strong pathway of support for patients between general practice and specialist DVA services.^17^ Although general practice engagement is an essential part of the IRIS programme, practices are facing competing challenges, including increasing scarcity of resources and recruitment challenges.^18^ This state of flux has worsened during the pandemic.^19^

**Table:** How this fits in

There was a shift to remote domestic violence and abuse (DVA) training in UK general practice during the COVID-19 pandemic. This qualitative study explored lessons learned from general practice and DVA specialist services regarding the transition to remote DVA IRIS (Identification and Referral to Improve Safety) training, with consideration of the impact on the overall DVA referral pathway between general practice and specialist DVA services. Although remote delivery can improve access to training for learners, this study has found concerns regarding a trade-off between the gain in access and a loss of learner engagement. Connectivity between general practice and specialist DVA services is important in establishing an effective referral pathway between both services.

Remote DVA training to general practice teams may continue beyond the pandemic. Remote education allows scalable, cost-effective and standardised learning, overcoming geographic and time constraints, and widening access.^20^^,^^21^ However, this may result in learner isolation, technical problems, and reduced visual cues that risk misunderstandings and represent a cognitive demand on trainers.^21^^,^^22^ Spontaneous conversation between trainers and learners, as well as between learners, may also be limited.^22^ Remote DVA training could have additional challenges because of the sensitive and emotionally challenging nature of its content, and participants may have lived experience of abuse. There is limited evidence on the impact of remotely delivering this content, and perspectives from those receiving and delivering training are important in deciding the future direction of remote DVA training.

The aim of this study was to understand the adaptations and impact of the shift to remote domestic abuse training during the COVID-19 pandemic in IRIS-trained general practices, by exploring perspectives of those delivering and receiving training. The broader impact of remote DVA training on the relationship between general practice and specialist DVA services is also considered. This study used the IRIS programme as a case study and is part of a larger study exploring the primary care response to DVA in the COVID-19 pandemic (PRECODE).^7^^,^^23^

## METHOD

There are multiple actors who support patients affected by DVA in general practice and in DVA specialist services. Any member of the whole general practice team can raise a DVA concern including GPs, nurses, and reception or administration staff; however, it is typically clinicians who refer patients to specialist DVA services where they are then supported by an advocate educator. Advocate educators work in partnership with clinical leads to deliver training together and connect with general practices.^11^ These connections help to establish a working relationship between general practice and specialist DVA services, which underpins the support for patients affected by DVA. For this reason, the current study considered perspectives from multiple standpoints, by conducting interviews with general practice professionals and IRIS advocate educators, as well as direct observation of training sessions.

Six IRIS-commissioned sites were selected using a multistage sampling framework. The first stage of sampling involved identification of 10 geographically diverse sample areas by IRISi regional managers (who support local implementation of IRIS), with six of these areas agreeing to participate. With the involvement of advocate educators in these six areas, and guided by IRISi regional managers, ‘snowballing’ was used to recruit individuals from general practices and advocate educators involved in the IRIS programme. Participants were approached using a range of methods including email and face to face. Data collection occurred between 20 April 2021 and 12 April 2022.

### Interviews with advocate educators and general practice staff

The qualitative study team conducted interviews with advocate educators from local domestic abuse agencies delivering the IRIS programme to explore their experience of delivering remote DVA training. Interviews were also conducted with general practice staff to understand their perspective of receiving training, with some GPs interviewed also having experience of co-delivering training with advocate educators. Prior to the interview, participants received an information sheet explaining the researcher’s reasons for conducting the research (see Supplementary Information S1). Interviews were conducted by the authors (a psychologist, a health services researcher, two academic GPs, a sociologist, and an academic clinical fellow). Interviews were semi-structured, used a flexible topic guide (included as Supplementary Information S2) and took place remotely, because of COVID-19 social distancing restrictions. The option of a telephone or online platform interview (Microsoft Teams or Zoom) was offered.

All participants provided informed consent that was audiorecorded before the interview, and contextual data were collected from participants in the interview. Only participants and researchers were present in interviews. No repeat interviews were carried out. The first author had a pre-existing professional relationship with a participant interviewed but this was not felt to affect overall findings.

Rapid qualitative analysis methods were used that were modified from McNall and Foster-Fishman^24^ and Vindrola-Padros *et al*.^25^ Data-driven inductive methods involved an interview summary using a rapid assessment procedure sheet, which was reviewed regularly in the research team to progress towards iterative data analysis.

All interviews were audiorecorded and encrypted, then transferred to an approved university transcription provider. Verbatim transcripts were anonymised. A framework approach was applied,^26^ which involved a first round of independent manual coding by the first author, with the subsequent development of a ‘working analytical framework’ (included as Supplementary Information S3) that was applied to all transcripts. The data were charted into a framework matrix in Microsoft Excel, which allowed connections to be made across the data and for key themes to be generated.

### Observation of training sessions

The use of observation methods, such as researchers being immersed in a setting where responders receive training, provided additional perspectives to those self-reported by participants in interviews. Remotely delivered IRIS training sessions were observed to evaluate enablers and barriers to training, content delivery, and receipt, and to consider the potential for relationship building between the general practices and IRIS advocate educators. Sessions were independently observed by six of the authors.

Observers joined the session as a participant on Microsoft Teams with the microphone silenced. During the session, the context and dynamics of training sessions, variations in training delivery, and demonstration of participants’ engagement were documented. An observation framework was applied, informed by Spradley,^27^ to record observations (included as Supplementary Information S4). The framework refers to the space and layout of the virtual setting, objects used, how time is managed, the actors involved, how they engage and communicate, and what activities they engage in.

The authors recognise that observations for remote training may have limitations compared with face-to-face settings, for example, the possibility of training participants having their cameras off, thereby having an impact on group interactions. However, the aim was discreet observation recording, analytical detachment, and minimising the influence of the researcher on the training environment, recognising the risk of potential observer bias and the impact the presence of an observer may have on primary data by influencing group elements.^28^

### Patient and public involvement

The study has been guided by a group of women advisers with lived experience and perspectives of accessing healthcare services for domestic abuse. Regular meetings helped to inform the research approach and overall application of research findings to support general practice in identifying and responding to domestic abuse.

## RESULTS

### Participants’ characteristics

#### Interviews

Twenty-one participants who were purposively sampled from NHS general practices and domestic abuse agencies delivering the IRIS programme from four regions in England and Wales were interviewed. Participant characteristics are described in Table 1. Seven advocate educators involved in training delivery and 14 general practice staff (three practice managers, eight GPs, and three reception team members) were interviewed. The average interview length was approximately 38 min.

**Table 1.** Interview participants' characteristics (*N* = 21)

**Characteristic**	***n* (%)**
**Type of healthcare professional**	
Advocate educator	7 (33)
GP	8 (38)
Practice manager	3 (14)
Reception team	3 (14)

**Gender**	
Female	20 (95)
Male	1 (5)

**Years spent working in sector**	
0–3	2 (10)
4–9	9 (43)
10–20	2 (10)
>20	3 (14)
Not specified	5 (24)

At least two GPs interviewed had experience in delivering DVA training. Training was one aspect of the interview topic guide and the most detailed perspectives on training were offered by 10 participants (seven advocate educators, two practice managers, and one GP). Advocate educators delivering training most commonly shared their views regarding the shift to remote training and this is reflected in the data below.

#### Training sessions

Eight IRIS training sessions delivered remotely by 11 advocate educators to general practice teams in England and Wales were observed. In total, 52 participants attended training across five groups of clinical staff (GPs, practice nurses, and general practice-based psychological wellbeing practitioners) and three groups of support staff (receptionists, administrators, and practice managers). Participant group sizes ranged from four to nine and were diverse in terms of age, gender, and ethnicity, with varied time in role (<1 year to >20 years). Training sessions were up to 2 h long for clinical staff and 1 h for support staff. Advocate educators sometimes delivered the sessions independently (five sessions) and sometimes in pairs (three sessions), if advocate educators preferred to share delivery and communication.

### Themes

Results were grouped into four themes identified from both the observation of the training sessions and the interviews, with the key themes below weaving across both datasets:

- constants and adaptations;
- the access–engagement trade-off;
- sensitive content in remote sessions; and
- is remote training here to stay?

Supplementary Table S1 highlights the main codes relevant to each theme. Supplementary Table S2 demonstrates how data from interviews and observations link to individual themes.

### Constants and adaptations

The shift towards remote training highlighted issues that previously existed in face-to-face training. These included challenges in scheduling DVA training with busy general practices facing competing demands. Major adaptations in the transition to remote training during the pandemic included changes in DVA training content, as well as adjusting to novel technology for those delivering and receiving training.

When scheduling training sessions with general practices, a variability in commitment among practices, pre-dating the pandemic, was reflected:

*‘There is always going to be some practices that are not as — they have never been as easy to get onboard as others. Some of them waiting for their update training they are, not resistant, but they are not good referrers, they don’t seem to be quite as onboard as some of the others.’*

(Advocate educator [AE]1, female, 4‒9 years in sector)

One advocate educator explained why arranging training with practices is essential for the relationship between general practice and specialist DVA services:

*‘… if they haven’t had the training then the communication is really poor. They just don’t have a clue who I am, what’s going on, who IRIS is, why the patient would be referred … I’m very confident in which surgeries I have that good rapport with ... ’*

(AE6, female, 4‒9 years in sector)

There have also been major adaptations in the shift to remote training delivery:

*‘We talked about the increase in referrals through the COVID 19 pandemic … that there had been more homicides due to domestic violence during the pandemic … But then a huge part of the training is exactly that of asking how to ask the questions of domestic abuse over phone and video consultation, how to do that safely. Because obviously women are at home and, their perpetrator could be right beside them or behind the computer, listening on the call.’*

(AE3, female, 0‒3 years in sector)

A focus on clinician empowerment was also highlighted by this GP who delivers training:

*‘I am giving doctors, clinicians, and nurses and things confidence that they need to actually get in there when they are on the phone and actually be curious and ask questions. And also work around issues of safety which are much more concerning if there is somebody in the background …’*

(GP1, female, >20 years in sector)

Adapting to novel technology was another major adjustment for those delivering and receiving training. When observing training sessions, the study team noticed technical problems across all sessions; screen camera and microphone faults occurred and some attendees were unable to connect.

Some advocate educators struggled with the new technology:

*‘Oh and I have had some disasters. I think once my electricity tripped and I got thrown out of the session and … You get back in, people understand and yes I was the one sweating, they were all quite fine waiting for me.’*

(AE1, female, 4‒9 years in sector)

Other advocate educators were surprised at how well they adapted:

*‘Yes, I was surprised by it. I don’t know maybe I was surprised because it was — I doubted my own abilities to deliver training online but yes, it is just I can’t sit in front of a screen and talk but I actually can.’*

(AE1, female, 4‒9 years in sector)

General practices receiving training also needed to adapt to novel, unfamiliar technology:

*‘This practice, they didn’t have the right equipment, they didn’t have enough webcams and laptops for everyone to be sat … This practice were really keen on doing it all together in one room with just one camera for all of them.’*

(AE2, female, 0‒3 years in sector)

Having multiple attendees in one room sharing a screen could be confusing for the advocate educator trainer:

*‘That was a nightmare because you just couldn’t hear anyone. You couldn’t see people. Rather than everyone being a little icon, it was one shot with everyone in. You had no idea who was saying anything. Usually, it flashes up with someone’s name when they speak. With this, you couldn’t tell if it was Doctor X or Doctor Y who was saying something.’*

(AE2, female, 0‒3 years in sector)

Among general practice staff receiving training, there was diverse technical ability:

*‘Speaking as a manager, we learn differently. So, I don’t mind learning on Teams. I’ve done quite a lot of training in the last 12 months on Teams, courses, that kind of thing. Or being on meetings like this. So, I’m used to it. A lot of the satellite receptionists, they’ve always done face-to-face training. They’ve never had to use Microsoft Teams. They’ve never had to use Zoom so a lot of them might find it a bit unusual and it might not sink in the same way.’*

(Practice manager 2, female, years in sector not specified)

### The access–engagement trade-off

A marked improvement in access to training was noted by advocate educators delivering training, with training including practice staff who otherwise would not have been able to attend, for example, participants joining from their home or on the school run.

One advocate educator was surprised at how effective remote delivery was in widening access to training:

*‘When people are at home with the children. You see them shooing them out of the room but they were still able to do the training so yes … it was quite phenomenal; it blew me away … Because I thought, “Wow we wouldn’t get these people at the training.”’*

(AE1, female, 4‒9 years in sector)

Remote training also allowed multiple practices to attend training at once:

*‘And we now can reach people from all 49 practices, so that training, where we were having different people from different practices, was a lot easier for us, a lot less time-consuming.’*

(AE4, female, 4‒9 years in sector)

However, with a gain in widened access came a perceived loss in overall engagement, having an impact on opportunities for learning. The study team noticed just under half the participants’ screen cameras were turned off during a training session and were only switched on for the initial introductions or when responding to, or asking, a question. This could affect the communication of non-verbal cues between trainer and participants, non-verbal cues that might indicate understanding, engagement, and emotional response that are important when discussing a subject such as DVA.

Participants were noticeably ‘multitasking’ during training: answering calls, eating lunch, and using the chat function to explain when they needed to attend to an urgent task and pause training. In contrast to previous face-to-face delivery that involved regular participant interaction, in remote sessions advocate educator trainers were the main speakers for the majority of the session time and participants were largely passive recipients.

It was observed that the advocate educators used a variety of techniques to engage trainees, for example, videos to illustrate key behaviours seen in DVA or ‘ice- breaker’ tasks. Despite these interventions there were still limits to engagement.

The trade-off in widening access to training at the expense of engagement was a concern for some advocate educators. One GP who is involved in training delivery explained that the loss of visual feedback from participants can be challenging for the facilitator:

*‘I would say that it feels very removed. You can’t make eye contact in the same way, you can’t read the responses that GPs are having, or clinicians are having to what they are being told, which is hard stuff.’*

(GP1, female, >20 years in sector)

At times the reduced engagement, compounded by technological challenges of training delivery, created stress and isolation for the facilitator:

*‘… with IRIS training, you play some videos and there is some kind of group work there too. And that was really hard to try and adapt to video because, you have to figure out how to kind of play the video and how can they hear it. Those little things really impact the stress levels when you’re trying to do training, and especially if no one is kind of — if no one has their screens on, it feels very odd to kind of just talk to yourself.’*

(AE3, female, 0‒3 years in sector)

### Sensitive content in remote sessions

The challenge of discussing a sensitive subject such as DVA in a remote setting was explored. In observation of training sessions, participants were reminded that the content could potentially be distressing and were reassured that they can simply switch off their screens/sound to ‘leave the room’ if they needed to. This is especially important given the possibility of training participants having lived experience themselves. However, this limited the trainer’s ability to ‘read the room’ and gauge a response from the audience, meaning that there was a risk trainees could be overwhelmed by the content, with no one close by to support them if needed.

Advocate educators acknowledged that this was challenging content to discuss in a virtual environment:

*‘That’s what the practice manager gave me as feedback is that perhaps, that wasn’t thought about enough. You know, that it is quite a lot of overwhelming information and really, there was no way to gauge that for me … because you can’t read the room literally.’*

(AE7, female, 4–9 years in sector)

One GP who delivers training shared concerns on establishing a safe remote learning environment:

*‘So, I think it is much easier to gauge how engaged your trainees, or the doctors you are teaching are, when you are in a room with them. And it is also much easier to set up a feeling of trust, and safety, people can ask whatever they want and there is no judgement* [more] *than it is possible to do remotely. I think that probably is the main issue … ’*

(GP1, female, >20 years in sector)

### Is remote training here to stay?

There was diversity of opinion among interview participants regarding the longevity of remote training. Remote training was viewed as an efficient option given the busy work schedules of practice staff, and for some advocate educators there was worry around reverting back to face-to-face training. On the other hand, some preferred a return to face-to-face training to enhance learning and improve absorption of information.

To their own surprise, one advocate educator felt remote training had significant benefits and actually could achieve a similar experience to face-to-face training:

*‘I am a real convert and I can’t believe I am even saying that. I can’t believe these words are coming out of my mouth … So, feedback-wise, I think it was good. It was as it always has been. They were asking the same sort of questions that they have always asked. So yes, I was actually really impressed with it, if I am honest. Seems to have gone really alright, yes.’*

(AE1, female, 4‒9 years in sector)

For another advocate educator, the thought of returning to face-to-face training caused worry:

*‘I have built up an anxiety around going back to doing that face-to-face because it has been easy to do it via Teams because I’m at home … For example, I can have my notes and it’s not so formal.’*

(AE6, female, 4‒9 years in sector)

Although acknowledging reduced engagement in remote training, one practice manager felt that remote training offered a significant advantage. It was felt to be a time-efficient option, complimentary to the work schedules of general practice staff:

*‘I think it works better in some ways … One, you haven’t got interaction with people there and then, but for time for people, they are not having to go places so they’ve got more time, then they can do the meetings, do the training, and then get on with their work.’* (Practice manager 1, female, >20 years in sector)

However, another practice manager expressed that they felt remote training was inferior to face-to-face training based on attendees absorbing less information:

*‘So, I think it’s different and not everybody will take on as much as they would if they were face-to-face, I don’t think. You can’t beat face-to-face.’*

(Practice manager 2, female, years in sector not specified)

## DISCUSSION

### Summary

In the transition to remote DVA training in general practices during the COVID-19 pandemic, this study found examples of successful adaptation to existing training content and delivery, which was well received by both general practice staff participants and trainers.

However, advocate educators found it challenging to schedule DVA training with busy practices and there were major adaptations in the content of DVA training, with a new and proactive focus on safely addressing potential or disclosed DVA in remote consultations alongside a need to adjust to novel technology. Variable technical ability among general practice staff and advocate educators was reported, and at times some general practices did not have the required technical infrastructure. This resulted in a halfway house of remote training delivery, with practice staff sharing rooms and screens, making it more difficult for advocate educators to connect with staff as individuals and complicating group communication. The reports from interviews were consistent with study team observations in training sessions.

The remote model widened access to training for those based away from the practice; however, there was a concurrent overall loss of participant engagement. Not meeting in person reduced important non- verbal cues for the trainer to pick up on and address. This is especially important given the sensitive content of training. Interaction between participants was variable and this potentially limited opportunities for learners to discuss emotionally challenging content as a group, including the sharing of experiences and reflections. Reduced learner engagement may have limited personal interactions between advocate educators and general practice staff, potentially weakening the working partnership that is critical in supporting DVA referrals from general practice to specialist DVA services.

This trade-off between access and engagement created mixed feelings among advocate educators delivering training: from surprise at the reach remote training could have, to stressful and isolating experiences for advocate educators facilitating the training. In the observations in this study it appeared that remote training was helpful in allowing general practice staff to subtly step away from sensitive content; but in interviews there were concerns that the loss of the ability to read the room in remote training made it more difficult to gauge training, and there was a risk of overwhelming the audience. This is of particular concern, given that staff may have personal DVA experience or know others who have lived experience.

Divergent opinions existed regarding the future of remote DVA training in general practice. Although some felt there was little to distinguish it from face-to-face training, others felt that the practice were likely to come away with less than they would have done in face-to-face settings.

### Strength and limitations

This is a novel area of research, and observation of training alongside the interviews conducted with those delivering and receiving training allowed the authors to triangulate key findings. Generalisability of findings is limited by the study only being performed in the context of the IRIS training, support, and referral programme. The general practices had specific guidance and training, as well as a referral pathway to a named specialist in a DVA service. The findings in the current study may be less generalisable to remote training that does not link to referral pathways or are one-off events. The study benefitted from the valuable perspectives of advocate educators who deliver training and general practice staff who predominantly receive training. The study had an imbalance in the number of advocate educators and general practice staff interviewed, and would benefit in future from more perspectives from the general practice teams receiving training. An additional limitation of the study was that a predominance of quotations came from two participants and the study would be strengthened by the inclusion of a greater range of perspectives.

### Comparison with existing literature

The findings in the current study provide insight into the experiences of delivering and receiving remote DVA training in general practice during the pandemic. They are consistent with previously reported advantages of remote training offering valuable inclusivity and widened access,^21^ alongside concerns regarding learner isolation, technical problems, and reduced engagement.^21^^,^^22^ Through the current study’s analysis of trainer perspectives, the study has contributed the additional insight of the experience of trainer isolation. There is limited research in this area, although it has been reported among teachers in a non-medical distance learning setting during the pandemic.^29^

Reduced engagement creates distance between advocate educators and general practice staff attending remote training. This is concerning because previous research has shown that personal interactions between GPs and advocate educators mean that advocate educators are viewed more positively.^17^ Therefore, reduced opportunity for personal interactions between advocate educators and general practice staff could negatively have an impact on the relationship between the general practice team and DVA specialist services. In contrast to some other types of training sessions, which may be a stand- alone or one-off session, the impact of these DVA training sessions extends beyond the session itself and can affect the ongoing partnership between general practice and DVA specialist services.

The current findings also raise questions regarding whether remote training is an appropriate forum to discuss sensitive and emotional content, such as DVA. More research is needed in this area, although concerns have already been raised about managing safeguarding in remote general practice consultations.^30^

### Implications for research and practice

Building strong relationships between general practice and DVA services is crucial in supporting patients affected by DVA. The IRIS training, support, and referral programme can help to achieve this, with DVA training to general practice staff being a key component. Although remote training widens access to sessions, the loss of engagement risks weakening the partnership between general practice and DVA services. The authors suggest a mixed DVA training model that includes remote information delivery alongside a supplemented face-to-face element. This would take advantage of the improved access from remote training while building on the general practice and DVA support service partnership in face-to-face settings.

In conclusion, further research is needed regarding remote training in primary care, including how to support those delivering training, achieving learner engagement, and the safeguarding of remote learners who are DVA survivors. The current findings have relevance for other organisations and services forming partnerships with primary care, for example, social prescribing and social services.
